# Prevalence and Associated Factors of Depression among Prisoners in Jimma Town Prison, South West Ethiopia

**DOI:** 10.1155/2018/5762608

**Published:** 2018-06-19

**Authors:** Zakir Abdu, Teshome Kabeta, Lamessa Dube, Workinesh Tessema, Mubarek Abera

**Affiliations:** ^1^Department of Psychiatry, Faculty of Public Health and Medical Sciences, Mettu University, Ethiopia; ^2^Department of Epidemiology, Faculty of Public Health, Institute of Health, Jimma University, Ethiopia; ^3^Department of Psychiatry, Faculty Medical Sciences, Institute of Health, Jimma University, Ethiopia

## Abstract

**Background:**

Mental disorder is one of the greatest challenges that current and future generations will face. Currently among all people suffering from depression, 85% of them live in low- and middle-income countries. Previous studies reported the global burden/prevalence of depression to be five to ten times higher among prisoners than the general population. However, the prevalence of depression among prisoners in our study area is not known.

**Objective:**

This study therefore aimed to assess the prevalence and associated factors of depression among prisoners in Jimma town in 2017.

**Method:**

A cross-sectional study design was employed on 332 prisoners selected by systematic random sampling method. Data was collected by a face to face interview using Beck Depression Inventory (BDI-II) scale. Data analysis was done using SPSS version 20.

**Result:**

The study revealed that 41.9% (*n* = 139) of participants among prisoners had depression. Having family history of mental illness (AOR = 6.05, 95% CI = 2.6, 13.8), having chronic physical illness (AOR = 2.87, 95% CI = 1.29, 6.41), having history of previous incarceration (AOR = 3.26, 95% CI = 1.02, 10.64), lack of job in the prison (AOR = 4.96, 95% CI = 2.09, 11.8), lifetime alcohol use (AOR = 3.61, 95% CI = 1.8, 7.26), thinking life to be a difficult one after release from prison (AOR = 2.07, 95% CI = 1.2, 3.6), having age between 21 and 25 years (AOR = 2.04, 95% CI = 1.06, 3.89), and having poor social support (AOR = 2.2, 95% CI = 1.27, 3.82) had significant association with depression in the fully adjusted final regression model.

**Conclusion:**

This study has shown that the prevalence of depression among prisoners was very high. Having family history of mental illness, having chronic physical illness, having previous incarceration, lack of job in prison, lifetime alcohol use, thinking life to be difficult one after release from prison, having age between 21 and 25 years old, and having poor social support were found to have an impact on the prevalence of depression.

## 1. Background

Mental disorder impose an enormous disease burden almost everywhere across the world [1]. It is one of the greatest challenges that the current and future generations will face [2]. Depression is the most common and severe but treatable mental disorder [3]. Out of people suffering from depression; 85% live in low- and middle-income countries [4]. When compared to the general population, worldwide, prisoners were five to ten times more likely to develop depression [5, 6].

The incidence of mental disorders among prisoners in the western countries reported that one from seven prisoners suffers from some type of psychiatric disorder and depression is the most one [6]. According to a systematic review from 24 western countries, 10.2% of male prisoners and 14.1% of female prisoners had depression [7]. From prison in United States (US), about 23% of state prisoners and 30% of jail imprisonments were found to have depression [8]. Prevalence of depression among prisoners in Asian country is more than two times higher than the western countries [9] but lower than the result in African [10]. According to studies conducted in different parts of Africa, 10.4% to 82.5% of the prisoners found to be depressed and the disorder is higher among females and young age groups [11–13]. One study from sub-Saharan country showed that the magnitude of depression among prisoners is 42.2% and the more affected group is the lower educational status and singles [10, 14]. Little is known in east Africa, and one study which is done recently in Ethiopia revealed that at least eight prisoners out of nineteen suffered from depression and it was associated with residence of prisoners [5].

Most studies done in prisons of different countries across the world revealed the following factors. Being female [6, 7, 15–18], older age [14, 19], performing work in prison (AOR = 0.49, CI 0.28–0.87), prisoners incarcerated repeatedly (AOR: 3.3, CI 1.7–6.3) [7, 20, 21], having past psychiatric history (OR: 1.9, CI 1.4–25) [21, 22], having family history of mental illness (AOR: 1.6, CI: (1.0–2.7)) [21], having history of previous substances used (AOR: 3, CI: 2.3, 4.0) [21], having poor social support [AOR: 0.62; CI 0.44, 0.89], and thinking life to be a difficult one after release from prison (AOR: 1.87, CI 1.30, 2.69) [5] were variables identified as they had association with depression.

## 2. Methods and Materials

An institutional based cross-sectional study was conducted from June 1 to June 15, 2017. All prisoners in Jimma town prison were included. Those prisoners who were not able to communicate because of any kind of illness were excluded from study. And those prisoners openly imprisoned were excluded.

To get maximum sample size, prevalence rate (*P*) of 50% was taken. Single population proportion formula was used to determine sample size at 95% CI and 5% marginal error.

(1) $$\begin{array}{c} n_{i}=\frac{{\left(z\overset{´}{\alpha }/2\right)}^{2}\times p\left(1-p\right)}{d^{2}}, \end{array}$$

where

*n*_*i*_ is initial sample size;
$\overset{´}{\alpha }$ is confidence interval (95%);
*p* is estimated proportion which was assumed to be 50% (0.5);
*d* is margin of sampling error tolerated (5%).
  (2) ni=1.962×0.51−0.50.052=3.8416×0.5×0.50.0025=384

The total number of prisoners in Jimma town Correctional Institution is 1460 which is less than 10,000. Using finite population correction formula the final sample size was

(3) $$\begin{array}{c} n_{f}=\frac{n_{i}}{1+n_{i}/N}, \end{array}$$

where

*n*_*f*_ is final sample size;
*n*_*i*_ is initial sample size calculated above (384);
*N* is total number of prisoners (1460).
  (4) nf=3841+384/1460⟹3841.259=305

Considering nonrespondent subjects and by adding 10% nonresponse rates the final number of the study subject became** 336.**

A systematic random sampling was done using the prisoners list and using the sampling interval size calculated using the formula

(5) $$\begin{array}{c} \frac{N}{n}=k, \end{array}$$

where *N* is the total population (1460) and *n* is sample size (336) while *k* is sampling interval size. *K* = (approximately 4). Therefore, every 4th prisoners on the list was selected. List prisoners from 1 to 1460 and then randomly select a number between 1 and 4 (e.g., “2”):

- 1st person selected = the 2nd on the list;
- 2nd person selected = 2 + 4, the 6th, etc.

Data was collected using structured questionnaire by face to face interview technique. BDI-II was used to screen the presence and absence of depression [23]. The Oslo 3-item social support scale was used to assess level of social support [24, 25].

### 2.1. Operational Definition

**Figure 1:**
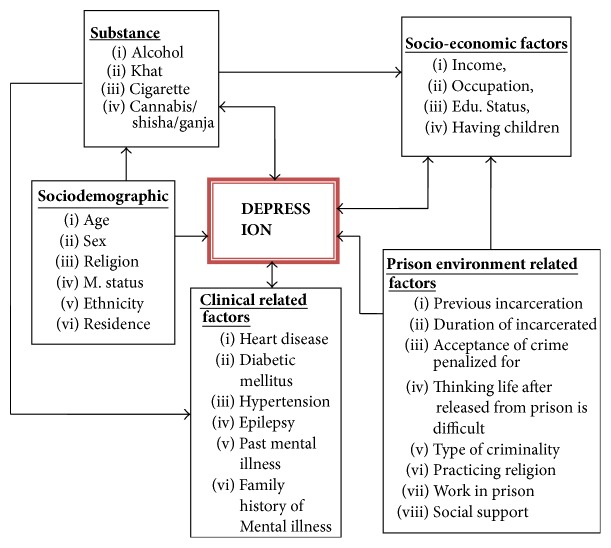
Conceptual framework of study on prevalence of depression and associated factors among prisoners in Jimma town Correctional Institution, South West Ethiopia, 2017 (source: developed by the principal investigator by reviewing literatures and scientific background).

Figure 1 shows the factors associated with depression among prisoners in Jimma town: sociodemographic (like age, sex, religion, m. status, ethnicity, residence), substance (like alcohol, khat, cigarette, cannabis/shisha/ganja), socioeconomic factors (like income, occupation, education status, having children), clinical related factors (like heart disease, diabetic mellitus, hypertension, epilepsy, past mental illness, family history of mental illness), and prison environment related factors (like previous incarceration, duration of incarceration, acceptance of crime penalized for, thinking life after released from prison is difficult, type of criminality, practicing religion, work in prison, social support).

**Figure 2:**
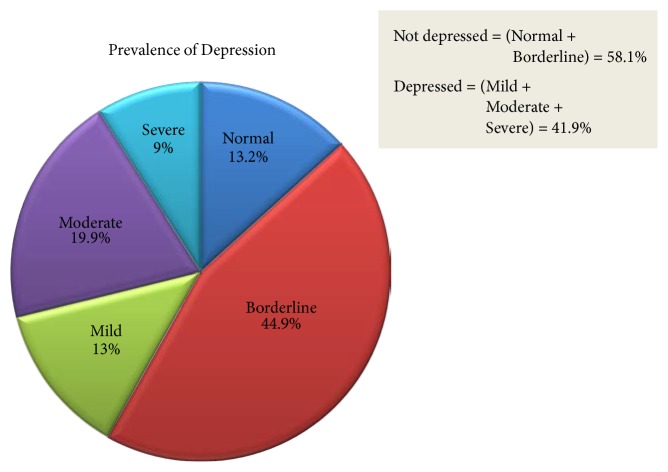
Levels of depression and its prevalence among prisoners in Jimma town Correctional Institution, June, 2017. *N* = 332.

Figure 2 shows the prevalence of depression: 43 (13.0%) had mild depression, 66 (19.9%) had moderate depression, and 30 (9.0%) had severe depression. About 44 (13.2%) of participants were without depression (normal) and 149 (44.9%) had borderline clinical depression.

According to BDI-II, depressed are participants who score 14 and more, while those who score 13 or lower are not depressed. From depressed participants, those who score 14–19 are mildly depressed, 20–28 are moderately depressed, and 29–69 are severely depressed.

Those* thinking life to be a difficult one after release from prison *are participants who believe that their life will not go as before being incarcerated when they become released from prison. It was assessed by a developed questionnaire which is a set of attitudes that generated a single score.

There are also participants with* chronic illness,* illness that can be managed but cannot be cured, and having a greater risk of developing depression, for example, heart diseases, diabetic mellitus, HIV/AIDS, past mental illness, etc. And the response for chronic illness is from self-report of the participants.

Those* accepting penalty for their crime* are participants who accept the fact that the reason why he/she was incarcerated was because of doing that crime. It was assessed by generating a single score.

### 2.2. Data Analysis

Data was analyzed using SPSS version 20.0. Binary and multivariable logistic regression analyses were employed. The variables which have a statistical significance association in the final multivariable analysis were identified on the basis of *p* values < 0.05 and AOR with 95% confidence intervals.

*Ethical Approval and Consent of Participants.* The study was conducted after ethical clearance is obtained from ethical review board of Jimma University (IHRPGC/761/2017). Confidentiality was ensured and all related questions they raised were answered. Participation was completely voluntary, with no economic or other motivation, and each participant signed written informed consent for their participation. Participants right to refuse or discontinue participation at any time they want was strictly respected.

## 3. Result

### 3.1. Sociodemographic and Economic Characteristics

From the total of 336 participants, 332 were interviewed. Among 332 prisoners participating, the majority were males, 311 (93.7%). The median age of the respondents was 26 years with an interquartile range of 12 years (Table 1).

### 3.2. Prison Related Environment Related

The study revealed that one in twelve (8.4%) of participants had history of previous incarceration. More than one-fourth (27.4%) of them stayed for <4 months in prison and the median time spent in prison was 10 months with an interquartile range of 20 months. About 7/16th (44.9%) thought that life might be difficult after release from prison. About one-fifth (19.0%) of respondents had work opportunities within the prison of whom 23 (6.9%) engaged in wood work. About half (50.9%) of respondents reported having poor social support

### 3.3. Clinical Factors Characteristics

The study revealed that about one-sixth (16%) of respondents had chronic physical illness. One in every eleven (9%) of the respondents had history of past mental illness (Table 2).

### 3.4. Prevalence of Depression

The prevalence of depression among prisoners in Jimma town prison over the past couple of weeks was found to be 41.9% (three in every seven) with a 95% CI and 36.7–46.7%. Of the total number of participants 13% had mild, 20% had moderate, and 9% had severe depression. About 44 (13.2%) of participants were found to have no depression (normal) and 149 (44.9%) had borderline clinical depression.

### 3.5. Factors Associated with Depression

Socioeconomic and prison environment characteristics of respondents like marital status, residence before incarceration, occupation, educational status, having children, duration of stay in prison, acceptance for the charge of crime, and opportunity to pray in prison did not show association with depression in the bivariate analysis. However, being female, being in the age group between 21 and 25 years, previous history of incarceration, thinking life to be a difficult experience after release from prison, lack of opportunity for job in the prison, type of criminality, presence of family member with mental illness, presence of chronic physical illness, past mental illness, and poor social support were associated with depression in the bivariate analysis (Table 3).

Regarding lifetime substance use bivariate analysis indicated that lifetime alcohol use, smoking cigarette, chewing khat and cannabis/shisha/ganja use were associated with depression and entered multivariate logistic regression model.

Multivariable logistic regression analysis revealed that having history of previous incarceration, having no opportunity for job in the prison, having family history of mental illness, having chronic physical illness, lifetime alcohol use, being in the age group between 21 and 25 years, having poor social support, and thinking life after released from prison is difficult had significant association with depression.

Accordingly, the odds of having depression among prisoners who were previously incarcerated were 3.26 times higher (AOR = 3.26, 95% CI = 1.02, 10.64) compared with those who were not previously incarcerated. The odds of having depression among prisoners who had no job in prison were 4.96 times higher (AOR = 4.96, 95% CI = 2.09, 11.80) than prisoners who had work in the prison. The odds of having depression were 6.05 times more (AOR = 6.05, 95% CI = 2.6, 13.80) among prisoners who had family member with mental illness as compared with prisoners who have no family members with mental illness. Prisoners who had chronic physical illness were 2.87 times (AOR = 2.87, 95% CI = 1.29, 6.41) more likely to develop depression than prisoners who have no chronic physical illness. Prisoners who were in the age group between 21 and 25 years were 2.04 times more likely (AOR = 2.04, 95% CI = 1.06, 3.89) to develop depression compared with prisoners in the age above 34 years. Prisoners who had poor social support were 2.2 times more likely (AOR = 2.2, 95% CI = 1.27, 3.82) to report depression than prisoners with strong social support. The odds of having depression among prisoners who were lifetime alcohol use were 3.61 times (AOR = 3.61, 95% CI = 1.80, 7.26) than prisoners who did not use alcohol in their lifetime. Prisoners who were thinking life after release from prison is difficult were 2.07 times more likely (AOR = 2.07, 95% CI = 1.2, 3.6) to develop depression when compared to those not thinking life after released from prison is difficult (Table 4).

## 4. Discussion

This study revealed the overall prevalence of depression among prisoners to be 41.9%. The result is lined with studies carried out in US 43% [20], Brazil 40% [15], Nigeria 42.2% [14], and Ethiopia and Amhara 43.8% [5]. However, it was higher than the study done in Brazil 9.9% [15]. In systematic review of 2300 prisoners in western countries the prevalence of depression was 10% among men and 12% among women [6], South Africa 10.4% [11], Iran 29% [9], and Nigeri 30.8% [19].

Results of this study showed that history of previous incarceration and depression has significant association. This might be due to prisoners developing depression as a result of repeated exposure for the prison environment [26]. The study done previously showed that prisoners performing work inside the prison were less depressed which is similar to the current result. This might be because they become adjusted to the difficult life of prison and also when they perform income-generating jobs they get income and because of this they become less depressed [21].

Results of this study showed that having family history of mental illness is significant predictors for depression. Previous studies support this result [21, 27]. This might be due to mental illness having genetic base [3, 27, 28]. From this study there was statistically significant relation between chronic physical illness and depression. The possible reason could be that depression is more commonly encountered in people who have chronic physical illness [28]. This study showed that prisoners who had poor social support were more likely to develop depression. The possible reason could be that depressed prisoners are likely to suffer in many domains of life and appear less likely to adapt to prison or life thereafter.

Results of this study revealed that prisoners aged 21–25 years old were more likely to develop depression than those above the age of 34 years. Previous study done in US [8] is in line with this result. The possible reason could be individual with this age group is more likely to report alcohol and other substance use before incarceration which might lead them to develop depression. Other literatures also reported as depression being more common in this age group [10]. Lifetime alcohol use and being depressed were significantly associated. A study done in Egypt [21] and US [29] was in line with the current result. The possible reason could be that prison is stressful environment which make prisoners distressed [19].

The result of this study showed that those prisoners who think life to be difficult after release from prison were more depressed. The possible reason could be feeling of inadequacy; as the prisoners worry about their future life, they are easily affected by depression [12].

### 4.1. Strength of the Study

Strength of this study is that the severity of depression in addition to the prevalence is determined. In addition, the tool we used is standardized and internationally recognized screening tool is used with high reliability to screen depression regardless of population characteristics.

### 4.2. Limitations

The study however could suffer from the following limitations. This study was cross-sectional study design; it did not allow establishing a temporal relationship between depression and associated factors. The study was institution based which could limit its generalizability to normal population and clinical setting. The other limitation of the data is regarding depression-related questions. Recall bias regarding lifetime substance use and question to assess factors like belief that life after prison will be difficult, acceptance of crime done, and chronic illness were asked by single generated questions which have no internationalized cutoff point or no Likert scale.

The areas yet to be studied in this population are socioeconomic and prison environment characteristics of respondents like marital status, duration of stay in prison, and lifetime substance use like cigarette, khat, and cannabis use have no association with depression. The other important things that are yet to be studied which we saw during this study but are not included in our study are life style conditions of prisoners in prison like food, place of sleep, recreational activity, and bullying. There are other areas that need investigation.

## 5. Conclusion and Recommendation

In conclusion, depression among prisoners was found to be significantly high. Prisoners who had strong social support and performing work in prison were less likely to have depression while those who had previous incarceration, had family history of mental illness, had chronic physical illness, had lifetime alcohol use, thought life after release from prison is difficult, and were between the age of 21 and 25 years were more likely to have depression. Routine screening and availing treatment in the prison may be of great importance. Future studies investigating the negative consequence of depression among prisoners might be relevant. Interventional studies to identify effective interventions modalities for depression among prisoners might also be relevant.

## Figures and Tables

**Table 1:** Background characteristics of study participants among prisoners in Jimma town prison, South West Ethiopia, June 2017 (*N* = 332).

Study variables	Frequency (*N*)	Percentage (%)
Gender		
Male	311	93.7
Female	21	6.3
Age		
18–20	82	24.7
21–25	78	23.5
26–34	90	27.1
>34	82	24.7
Marital status		
Married	132	39.8
Single	180	54.2
Other^**∗**^	20	6.0
Ethnicity		
Oromo	218	65.4
Amhara	51	15.4
Dawuro	34	10.2
Other^**∗∗**^	29	8.7
Religion		
Muslim	189	56.9
Orthodox	110	33.2
protestant	33	9.9
Residence before imprisonment		
Rural	115	34.6
Urban	217	65.4
Monthly income (ETB)^a^		
<500	110	33.1
501–1000	65	19.6
1001–2500	75	22.6
>2501	82	24.7
Educational status		
Not educated	47	14.2
1–8 Grade	176	53.0
9–12 Grade	81	24.4
Higher education	28	8.4
Occupational before incarceration		
Employed	154	46.4
Unemployed	178	53.6
Having children		
Yes	134	40.4
No	198	59.6

^**∗**^Widowed, separated, and divorced. ^**∗∗**^Kefa, Gurage, Tigre, and Yem. ^a^Current currency 1$ = 23.35ETB.

**Table 2:** Clinical factors and Lifetime substance use characteristics of study participants among prisoners in Jimma town prison, South West Ethiopia, June 2017 (*N* = 332).

Study variables	Frequency (*N*)	Percentage (%)
Family member with mental illness		
Yes	57	17.2
No	275	82.8
Chronic physical illness^*∗*^		
Yes	53	16.0
No	279	84.0
Past mental illness		
Yes	30	9.0
No	302	91.0
Alcohol Use (lifetime)		
Yes	72	21.7
No	260	78.3
Khat Use (lifetime)		
Yes	56	16.9
No	276	83.1
Cigarette smoking (lifetime)		
Yes	152	45.8
No	180	54.2
Cannabis/shisha/ganga use (lifetime)		
Yes	22	6.6
No	310	93.4

^*∗*^Heart disease, hypertension, diabetes mellitus, epilepsy, HIVV/AIDS, and asthma.

**Table 3:** Factors associated with depression by bivariate logistic regression among prisoners in Jimma town prison, South West Ethiopia, June 2017 (*N* = 332).

Study variables	Depression		COR (95% CI)	*p*-value
	Yes *N* (%)	No *N* (%)		
*Gender*				
Female	14 (66.7)	7 (33.3)	2.97 (1.17, 7.58)	0.022
Male	125 (40.2)	186 (59.8)	1	
*Age (year)*				
18–20	29 (35.4)	53 (64.6)	0.69 (0.41, 1.17)	0.17
21–25	43 (55.1)	35 (44.9)	**2.02 (1.21, 3.38)**	**0.007**
26–33	36 (40.0)	54 (60.0)	0.9 (0.55, 1.47)	0.64
>34	31 (37.8)	51 (62.2)	1	
*Marital status *				
Married	55 (41.8)	77 (58.3)	1	
Single	71 (39.4)	109 (60.6)	0.805 (0.519, 1.25)	0.33
Other	13 (65.0)	7 (35.0)	2.741 (1.06, 7.062)	0.037
*Ethnicity*				
Oromo	94 (43.1)	124 (56.9)	1	
Amhara	22 (43.1)	29 (56.9)	1.06 (0.582, 1.943)	0.842
Dawuro	10 (29.4)	24 (70.6)	0.546 (0.252, 1.182)	0.125
Other	13 (44.8)	16 (55.2)	1.14 (0.53, 2.45)	0.735
*Religion *				
Muslim	80 (42.3)	109 (57.7)	1	
Orthodox	47 (42.7)	63 (58.1)	1.05 (0.66, 1.67)	0.82
Protestant	12 (36.4)	21 (63.6)	0.774 (0.367, 1.631)	0.5
*Educational status*				
Illiterate	24 (17.3)	23 (11.9)	0.986 (0.414, 2.35)	0.974
1–8 grade	68 (48.9)	108 (56.0)	1.37 (0.667, 2.821)	0.391
9–12 grade	35 (25.2)	46 (23.8)	0.828 (0.485, 1.412)	0.487
Higher education	12 (8.6)	16 (8.3)	1	
*Occupation*				
Unemployed	76 (54.7)	102 (52.8)	1.076 (0.695, 1.667)	0.742
Employed	63 (45.3)	91 (47.2)	1	
*Residence before incarceration*				
Rura	46 (40.0)	69 (60.0)	1	
Urban	93 (42.9)	124 (57.1)	1.125 (0.71–1.78)	0.616
*Having children *				
Yes	53 (38.1)	86 (61.9)	1	
No	81 (42.0)	112 (58.0)	0.852 (0.546, 1.331)	0.482
*Previous incarceration*				
Yes	23 (82.1)	5 (17.9)	**7.45 (2.758**–**20.152)**	**<0.001**
No	116 (38.2)	188 (61.8)	1	
*Duration of stay in prison (in month)*				
<4	35 (38.5)	56 (61.5)	1	
5–10	38 (45.2)	46 (54.8)	1.2 (0.73–1.98)	0.469
11–24	37 (48.1)	40 (51.9)	1.39 (0.83–2.31)	0.21
>25	29 (36.2)	51 (63.8)	0.73 (0.43–1.23)	0.243
*Thinking life after released from prison is difficult*				
Yes	77 (51.7)	72 (48.3)	**2.08 (1.34**–**3.25)**	**0.001**
No	62 (33.9)	121 (66.1)		
*Acceptance of criminality*				
No	91 (43.3)	119 (56.7)	1.179 (0.748–1.857)	0.48
Yes	48 (39.3)	74 (60.7)	1	
*Criminal type*				
Robbery	67 (46.5)	77 (53.5)	1	
Rape	8 (23.5)	26 (76.5)	0.35 (0.15, 0.83)	0.08
Corruption	6 (27.3)	16 (72.7)	0.43 (0.16, 1.16)	0.90
Murderer	45 (45.0)	55 (55.0)	0.94 (0.56, 1.57)	0.47
Other^*∗*^	13 (40.6)	19 (59.4)	0.78 (0.36–1.71)	0.74
*Pray in prison *				
No	36 (75.0)	12 (25.0)	**5.27 (2.627**–**10.581)**	**<0.001**
Yes	103 (36.3)	181 (63.7)	1	
*Having work in prison*				
No	127 (47.2)	142 (52.8)	**3.8 (1.94, 7.45)**	**<0.001**
Yes	12 (19.0)	51 (81.0)	1	
*Family with mental illness*				
Yes	43 (75.4)	14 (24.6)	**5.73 (2.98, 10.99)**	**<0.001**
No	96 (34.9)	179 (65.1)	1	
*Chronic physical illness*				
Yes	36 (67.9)	17 (32.1)	**3.62 (1.94, 6.77)**	**<0.001**
No	103 (36.9)	176 (63.1)	1	
*Past mental illness*				
Yes	25 (83.3)	5 (16.7)	**8.24 (3.07, 22.14)**	**<0.001**
No	114 (37.7)	188 (62.3)	1	
*Level of social support*				
Strong	13 (26.0)	37 (74.0)	1	
Moderate	38 (33.6)	75 (66.4)	**0.157 (0.09**–**0.277)**	**<0.001**
Poor support	88 (52.1)	81 (47.9)	**16 (9.13**–**28.27)**	**<0.001**
*Alcohol use (life time)*				
Yes	50 (69.4)	22 (30.6)	**4.37 (2.49, 7.67)**	**<0.001**
No	89 (34.2)	171 (65.8)	1	
*Khat use (lifetime)*				
Yes	82 (53.9)	70 (46.1)	**2.53 (1.61, 3.95)**	**<0.001**
No	57 (31.7)	123 (68.3)	1	
*Cigarette smoking (lifetime)*				
Yes	37 (66.1)	19 (33.9)	**3.32 (1.81, 6.08)**	**<0.001**
No	102 (37.0)	174 (63.0)	1	
*Cannabis/shisha/ganja use(life time)*				
Yes	17 (81.0)	4 (19.0)	**6.58 (2.16, 20.03)**	**0.001**
No	122 (39.2)	189 (60.8)	1	

^*∗*^Illegal trading, arson, related to government, and illegal marriage.

**Table 4:** Factors associated with depression by multivariable logistic regression among prisoners in Jimma town prison, South West Ethiopia, June 2017 (*N* = 332).

Study variables	Depression		AOR (95% CI)	*p*-value
	Yes *N* (%)	No *N* (%)		
*Gender*				
Female	14 (66.7)	7 (33.3)	2.35 (0.76, 7.25)	0.13
Male	125 (40.2)	186 (59.8)	1	
*Age (year)*				
18–20	29 (35.4)	53 (64.6)	1.04 (0.45, 2.40)	0.93
21–25	43 (55.1)	35 (44.9)	**2.04 (1.06, 3.89)**	**0.03**
26–33	36 (40.0)	54 (60.0)	0.93 (0.47, 1.81)	0.83
>34	31 (37.8)	51 (62.2)	1	
*Previous incarceration*				
Yes	23 (82.1)	5 (17.9)	**3.26 (1.02, 10.64)**	**0.05**
No	116 (38.2)	188 (61.8)	1	
*Thinking life after released from prison is difficult*				
Yes	77 (51.7)	72 (48.3)	**2.07 (1.2, 3.6)**	**0.009**
No	62 (33.9)	121 (66.1)	1	
*Criminal type*				
Robbery	67 (46.5)	77 (53.5)	1	
Rape	8 (23.5)	26 (76.5)	0.40 (0.14, 1.12)	0.08
Corruption	6 (27.3)	16 (72.7)	0.93 (0.28, 3.02)	0.90
Murderer	45 (45.0)	55 (55.0)	1.26 (0.66, 2.40)	0.47
Other^*∗*^	13 (40.6)	19 (59.4)	0.85 (0.33, 2.18)	0.74
*Having work in prison*				
No	127 (47.2)	142 (52.8)	**4.96 (2.09, 11.80)**	**<0.001**
Yes	12 (19.0)	51 (81.0)	1	
*Family with mental illness*				
Yes	43 (75.4)	14 (24.6)	**6.05 (2.60, 13.80)**	**<0.001**
No	96 (34.9)	179 (65.1)	1	
*Chronic physical illness*				
Yes	36 (67.9)	17 (32.1)	**2.87 (1.29, 6.41)**	**0.01**
No	103 (36.9)	176 (63.1)	1	
*Past mental illness*				
Yes	25 (83.3)	5 (16.7)	2.87 (0.84, 9.80)	0.09
No	114 (37.7)	188 (62.3)	1	
*Level of social support*				
Strong	13 (26.0)	37 (74.0)	1	
Moderate	38 (33.6)	75 (66.4)	1.07 (0.43, 2.66)	0.88
Poor support	88 (52.1)	81 (47.9)	**2.20 (1.27, 3.82)**	**0.005**
*Alcohol use (life time)*				
Yes	50 (69.4)	22 (30.6)	**3.61 (1.80, 7.26)**	**<0.001**
No	89 (34.2)	171 (65.8)	1	
*Khat use (lifetime)*				
Yes	82 (53.9)	70 (46.1)	1.71 (0.95, 3.07)	0.07
No	57 (31.7)	123 (68.3)	1	
*Cigarette smoking (lifetime)*				
Yes	37 (66.1)	19 (33.9)	1.78 (0.77, 4.10)	0.17
No	102 (37.0)	174 (63.0)	1	
*Cannabis/shisha/ganja use (life time)*				
Yes	17 (81.0)	4 (19.0)	2.08 (0.47, 9.24)	0.33
No	122 (39.2)	189 (60.8)	1	

^*∗*^Illegal trading, arson, related to government, and illegal marriage.
